# GA_3_ Treatment Delays the Deterioration of ‘Shixia’ Longan during the On-Tree Preservation and Room-Temperature Storage and Up-Regulates Antioxidants

**DOI:** 10.3390/foods12102032

**Published:** 2023-05-17

**Authors:** Tao Luo, Xiaolan Lin, Tingting Lai, Libing Long, Ziying Lai, Xinxin Du, Xiaomeng Guo, Liang Shuai, Dongmei Han, Zhenxian Wu

**Affiliations:** 1 College of Horticulture, South China Agricultural University, Guangzhou 510642, China; luotao0502@scau.edu.cn (T.L.); linxl4595@stu.scau.edu.cn (X.L.); ltt7059@stu.scau.edu.cn (T.L.); longlibing@stu.scau.edu.cn (L.L.); laiziying@stu.scau.edu.cn (Z.L.); xinxindu@stu.scau.edu.cn (X.D.); 31000462@scau.edu.cn (X.G.); 2 Guangdong Provincial Key Laboratory of Postharvest Science of Fruits and Vegetables, Engineering Research Center of Southern Horticultural Products Preservation, Ministry of Education, Guangzhou 510642, China; 3 Key Laboratory of Biology and Genetic Improvement of Horticultural Crops (South China), Ministry of Agriculture and Rural Affairs, College of Horticulture, South China Agricultural University, Guangzhou 510642, China; 4 College of Chemistry and Food Science, Nanchang Normal University, Nanchang 330032, China; shuailiang1212@hzxy.edu.cn; 5 Institute of Fruit Tree Research, Guangdong Academy of Agricultural Sciences, Key Laboratory of South Subtropical Fruit Biology and Genetic Resource Utilization, Ministry of Agriculture, Guangzhou 510640, China; handongmei@gdaas.cn

**Keywords:** longan (*Dimocarpus longan* Lour.) fruit, preharvest GA_3_ treatment, antioxidants, total flavonoid content, total phenolic content, storability

## Abstract

Gibberellic acids had been proven to improve the fruit quality and storability by delaying deterioration and maintaining the antioxidant system. In this study, the effect of GA_3_ spraying at different concentrations (10, 20, and 50 mg L^−1^) on the quality of on-tree preserved ‘Shixia’ longan was examined. Only 50 mg L^−1^ GA_3_ significantly delayed the decline of soluble solids (22.0% higher than the control) and resulted in higher total phenolics content (TPC), total flavonoid content (TFC), and phenylalanine ammonia-lyase activity in pulp at the later stages. The widely targeted metabolome analysis showed that the treatment reprogrammed secondary metabolites and up-regulated many tannins, phenolic acids, and lignans during the on-tree preservation. More importantly, the preharvest 50 mg L^−1^ GA_3_ spraying (at 85 and 95 days after flowering) led to significantly delayed pericarp browning and aril breakdown, as well as lower pericarp relative conductivity and mass loss at the later stages of room-temperature storage. The treatment also resulted in higher antioxidants in pulp (vitamin C, phenolics, and reduced glutathione) and pericarp (vitamin C, flavonoids, and phenolics). Therefore, preharvest 50 mg L^−1^ GA_3_ spraying is an effective method for maintaining the quality and up-regulating antioxidants of longan fruit during both on-tree preservation and room-temperature storage.

## 1. Introduction

Longan (*Dimocarpus longan* Lour.), which originates from China and belongs to the Longan genus of Sapindaceae, is an important subtropical and globally consumed fruit [1,2]. Sweetness is an important commodity property of mature longan fruit, as its soluble solids content (SSC) usually exceeds 20% [3]. More importantly, except being used as an important specialty fruit, longan fruit has been used as traditional Chinese medicine for thousands of years, due to the abundant bioactive components (e.g., polyphenols, flavonoids, alkaloids, polysaccharides) in pericarp, pulp, and seed [4,5,6]. These components in longan showed a broad range of health effects and activities, such as anti-tumor, antioxidant, anti-α-glucosidase, anti-α-amylase, and immunological activities [5,6]. However, longan fruit is a perishable fruit because its deterioration, mainly characterized as pericarp browning and aril breakdown, quickly occurs within a few days under room-temperature storage [1].

Doses of chemical, physical, and biological treatments have been reported to control postharvest decay, reduce pericarp browning, and reduce aril breakdown, thus extending storage life of longan fruit [1,7]. Among these treatments, due to their long-term effectiveness and convenience, SO_2_ fumigation and fungicide treatments are still widely applied in China’s domestic markets and other major producer countries to retard deterioration of longan fruit [7,8]. However, due to the harmfulness to the environment and human health caused by the residual sulfite, environmentally friendly and safe preservation methods are urgently needed to replace fungicide treatments and SO_2_ fumigation on longan fruit [1,8]. Among these alternatives, appropriate hormone treatments were proved to be effective to regulate fruit quality and storability. Phytohormones have attracted much attention in postharvest technology in the recent years, due to their key regulation capabilities in fruit physiology and biochemistry, such as stimulating response to biotic stress, delaying senescence, maintaining cell integrity, and, especially, enhancing the antioxidant system [9].

Gibberellic acids (GAs, mainly GA_3_) enhance earlier blooming, promote cell division, create seedless fruit, and increase fruit size and yield [10]. Due to their antagonistic effects with senescence-related hormones, such as ETH and ABA, GAs have been widely used for extending the shelf life of horticultural products [9]. Montero et al. (1998) found that the exogenous GA_3_ (30 μg L^−1^) spraying on the young plants enhanced the anthocyanin content and PAL activity in strawberry (*Fragaria ananassa* cv. Chandler) fruit [11]. Southwick et al. (2000) reported that preharvest GA spraying at 31 or 62 mg L^−1^ increased the firmness of ‘French’ prune *(Prunus domestica* L.) in continuous experiments of three years (1997, 1998 and 1999) [12]. The application of 100 mg L^−1^ GA_3_ (twice, 3 days before and 15 days after anthesis) on clusters induced seedless grape berry, enhanced berry size, and accelerated the development of berries, resulting in earlier ripening of seedless berries. However, this treatment resulted in higher TPC, TFC, and antioxidant activities in leaf, stem, and tendril, but remarkably decreased TPC, TFC, and antioxidant activities in berry skin and flesh [13]. It was observed that treatment with 2.7% GA to the carpopodium on Day 28 after blooming reduced the lignin biosynthesis of pear fruit by down-regulating the expression of genes which encode phenylalanine ammonia-lyase (PAL), 4-coumarate: coenzyme A ligase (4CL), cinnamyl alcohol dehydrogenase (CAD), and peroxidase (POD) [14]. It was also found that the combined treatments of CPPU and GA_3_ on green mature banana significantly suppressed fruit softening, delayed peaks of respiration and ethylene production rates, retarded chlorophyll degradation, delayed the increase of soluble reducing sugars, and reduced the loss of ascorbic acid and total phenols during storage [15]. The ‘0900 Ziraat’ cherry sprayed with 50 mg L^−1^ benzyladenine (BA) combined with 50 mg L^−1^ GA_4+7_ at yellow color stage was significantly larger and firmer, and showed higher SSC than the untreated fruits at harvest and during cold storage [16]. Another document showed that 30 or 60 mg L^−1^ GA_3_ spraying on cherry at straw-yellow stage significantly enlarged the fruit size, but generally led to lower SSC, as well as significantly lower TPC, total anthocyanin content, and total antioxidant capacity [17]. Both of the 30 mg L^−1^ GA_3_ and 30 mg L^−1^ GA_4+7_ spraying on the whole canopies of ‘English Morello’ sour cherry trees at 58 days after anthesis (DAA) delayed leaf senescence, and GA_3_ treatment had a better positive effect on the fruit size in two consecutive years [18]. The foliar application of 50 mg L^−1^ GA_3_ plus 10 mg L^−1^ CPPU on “Washington Navel” orange trees at 15, 30, and 45 days after full bloom gave the highest fresh weight, length, yield, and SSC (%) of fruit, while 50 mg L^−1^ GA_3_ plus 50 mg L^−1^ BA resulted in the greatest diameter, content of chlorophyll, TA (%), and vitamin C (VC) content, as well as decreased splitting (%) and carotene content in fruit [19]. Jujube fruit sprayed with 15 mg L^−1^ GA_3_ (on-tree at 2 different times: 3 and 2 weeks before harvest) showed significantly higher weight, width, hue angle, and firmness values, but significantly lower L* values than the control fruits. More importantly, jujube fruit treated with 15 mg L^−1^ GA_3_ and 15 mg L^−1^ GA_3_ plus 1% Parka (containing 7.5% stearic acid, 5% cellulose, and 1% calcium) showed a significantly lower respiration rate and SSC, but higher titratable acidity (TA), VC, and total phenolics [20]. The above reports indicated that the application of GAs had a significant effect on fruit ripening, quality (especially content of antioxidants), and storability, but it was worth noting that the effect of GA_3_ application depended on the fruit species, variety, application method, dose, treatment time, and organs.

For longan fruit, the effect of preharvest application of phytohormone on quality, antioxidants, and storability had not been investigated. Treatment with 10 mg L^−1^ GA_3_ at 5 DAA increased the weight, volume, longitudinal and traverse diameter, and the weight of the aril of ‘Shixia’ longan fruit and led to earlier accumulation of sugars (mainly sucrose, glucose, and fructose), but did not affect the proportion and content of sugars at harvest [21]. Similarly, spraying with 30 mg L^−1^ GA_3_, ABA, or IAA at 10 DAA did not alter the sugar content of mature longan fruit, but did accelerate the sugar accumulation before ripening stages [22]. However, the effect of GA_3_ treatment on quality and antioxidants at harvest, as well as storability of longan fruit, was still not investigated. Sugar receding, i.e., a sharp decrease in SSC in pulp, leads to a fast decline in sweetness and deteriorates the edible quality of on-tree preserved longan fruit [3]. In this study, we reported an effective preharvest GA_3_ spraying, through our previous screening assay, to delay the sugar receding of ‘Shixia’ longan fruit during on-tree stages. Further, the effect of GA_3_ treatment on the quality, the content of antioxidants, and enzymatic activity in longan pulp, as well as the storability of longan fruit, was analyzed. A widely targeted metabolome was performed to evaluate the effect of this treatment on the primary and secondary metabolites in longan pulp. These results will benefit the development of new methods to delay sugar receding, maintain the nutrition and quality during on-tree preservation, and improve the storability of longan fruit.

## 2. Materials and Methods

### 2.1. Preharvest Spraying Treatments and Fruit Harvest

The fruits from three longan trees (*D. longan* cv. Shixia, 15 years old), which had even canopy size and fruit load, were selected for treatments. The spraying experiments were carried out in the two consecutive years (2020 and 2021) in the same commercial orchard in Huidong (Guangdong province, China). In the year 2020, the preharvest spraying of 0 (control), 10, 25, and 50 mg L^−1^ GA_3_ (dissolved in 1 mL ethanol and then diluted with 999 mL ddH_2_O) on fruits was performed five times, at 89, 96, 103, 110, and 120 days after flowering (DAF); the time of full bloom was about 10 April 2020. Three bunches of fruits from each tree were selected for each spraying treatment. About 500 mL solution was used for spraying each bunch of fruits (about 60–80 fruits). For each treatment, 9 fruits from each bunch were randomly selected and harvested before each spraying, at 96, 103, 110, and 120 DAF. Finally, the rest fruits from each bunch were harvested at 130 DAF. The fruits harvested from the same tree were considered as one replication.

In the year 2021, the preharvest spraying of 0 (control), 10, 50, and 150 mg L^−1^ GA_3_ (dissolved in 1 mL ethanol and then diluted with 999 mL ddH_2_O) on fruits was performed five times at 85, 95, 105, and 115 DAF (the time of full bloom was about 10 April 2021). Three bunches of fruits from each tree were randomly selected for each spraying treatment. Another 3 fruit bunches from each tree were selected and subjected to spraying (50 mg L^−1^ GA_3_ or control) 2 times at 85 DAF and 95 DAF, and then the fruits were harvested at 105 DAF and used for the storage experiment. The fruits harvested from the same tree were considered as one replication.

### 2.2. Determination of Total Soluble Solids (TSS) Content, TFC, and TPC

The TSS content (%) was measured by a Brix refractometer (PAL-BX/ACID F5, ATAGO Co., Ltd., Tokyo, Japan). The total flavonoid content (mg g^−1^) and total phenolic content (mg g^−1^) of longan pericarp and pulp were measured according to the previously reported method [23,24]. The ethanolic extract used for determination of TFC and TPC was prepared by homogenizing 0.1 g of frozen pericarp or 0.5 g of frozen pulp, which was extracted using 3 mL 80% ethanol and centrifuged at 5000× *g* for 5 min. After being extracted three times, the ethanolic extracts was combined in a volumetric flask and adjusted to 10 mL using 80% ethanol [24]. The TFC was determined by the Folin–Ciocalteu method [23], while the TPC was measured using a modified colorimetric method, as previously described [24].

### 2.3. Measurement of the Activity of Phenylalanine Ammonia-Lyase (PAL) and Peroxidase (POD)

The PAL activity was assayed according to the reported method [25]. The frozen pulp or pericarp was ground into powder by liquid nitrogen. Then, 0.1 g pericarp (or 0.25 g pulp) was weighed and mixed with 0.75 mL Tris-HCl buffer (50 mM, pH 8.9), containing 2% polyvinylpyrrolidone (*w/v*), 2 mM EDTA, and 5 mM β-mercaptoethanol), in a 2 mL tube inside an ice-bath. After being fully vortexed, the sample was subjected to centrifugation at 12,000× *g* and 4 °C for 30 min. The supernatant was used for determining PAL activity. The reaction system consisted of 0.05 mL supernatant, 0.2 mL 50 mM Tris-HCl buffer, and 0.05 mL 20 mM phenylalanine. The mixture was incubated for 30 min at 37 °C, and then the reaction was stopped by adding 30 μL 6 M HCl. A change of 0.01 in the absorbance at 290 nm per minute was recorded as one unit of PAL enzyme activity. The result was expressed as U g^−1^ FW.

The POD activity was measured by a change in absorbance at 470 nm caused by production of tetraguaiacol from guaiacol in the presence of H_2_O_2_ [26]. The frozen pulp or pericarp was ground into powder by liquid nitrogen. Then, 0.2 g pulp or 0.1 g pericarp was weighed and mixed with 1 mL PBS buffer (50 mM, pH 5.5, containing 2% polyvinylpyrrolidone, *w/v*) in a tube inside an ice-bath. After being fully vortexed and extracted in a sonicator for 10 min, the sample was subjected to centrifugation (15,000× *g*) for 20 min at 4 °C. The supernatant was used for determining POD activity. Supernatant volume of 0.03 mL was mixed with 150 μL reaction solution, which was prepared by mixing 3 mL 50 mM PBS buffer, 1 mL 2% H_2_O_2_, and 1 mL 50 mM guaiacol. The reaction mixture was incubated for 10 min at 35 °C, and then the reaction was stopped by adding 60 μL 20% (*w/v*) trichloroacetic acid. A change of 0.01 in the absorbance per minute was recorded as one unit of POD enzyme activity. The result was expressed as U g^−1^ FW.

### 2.4. Widely Targeted Metabolome Analysis of On-Tree Preserved Longan Pulp Samples

The frozen pulp was crushed by zirconia beads in a MM 400 mixer mill (Retsch, Shanghai, China) for 1.5 min at 30 Hz. Powdered sample (100 mg) was fully mixed with 1.0 mL 70% aqueous methanol and extracted overnight at 4 °C. Each sample was vortexed three times during the period to increase the extraction efficiency. After being centrifuged at 10,000× *g* for 10 min, the supernatant was collected and filtered through a Carbon-GCB SPE Cartridge (250 mg, 3 mL, CNWBOND, ANPEL). Then, samples were filtered (SCAA-104, 0.22 μm pore size; ANPEL, http://www.anpel.com.cn//Products.html?id=118 (accessed on 22 July 2021) before LC–MS/MS analysis. Multiple reaction monitoring (MRM) of triple quadrupole mass spectrometry was employed to quantify each metabolite [27]. The parameters of LC–MS/MS analysis and data processing (orthogonal partial least squares discriminant analysis, OPLS-DA [28]) were set according to the previously reported method [2]. Based on the OPLS-DA analysis and significance test, the metabolites with variable importance in projection (VIP) value ≥1 and 1.5 fold-change (FC) (or 2.0-FC) (the Control vs. GA_3_-treated sample, *t*-test, *p*-value < 0.05) were identified as the significantly differently accumulated metabolites (DAMs). The cpd_id of each identified metabolite was searched by manually examining its exact mass in the KEGG COMPOUND database (https://www.kegg.jp/kegg/compound/ (accessed on 1 August 2021)). The KEGG enrichment analysis of DAMs was performed by the web tool Metabolites Biological Role (MBROLE) 2.0 [29].

### 2.5. Postharvest Treatment, Storage, and Sampling

More than 500 (control or 50 mg L^−1^ GA_3_-treated) fruits with no disease and no damage were selected and dipped in 500 mg L^−1^ prochloraz solution for 2 min. The fruits were then dried at room temperature for 20 min. After that, the fruits were packed into fruit trays (about 25–30 fruits per tray) with 0.01 mm-thick polyethylene films and stored at room temperature (RT, 25 ± 1 °C; 85% relative humidity).

The sampling of the RT-stored fruits was performed at 0, 2, 4, 6, 8, and 10 days after harvest (DAH). The pericarp and pulp of fruits were separated. The inner surface of each pericarp was examined, and then the pericarp browning index as well as the aril breakdown index were evaluated (see Section 2.6). The whole collected pericarp and one half of the collected pulp were immediately frozen in liquid nitrogen, ground, and stored at −80 °C until used. The other half of the pulp was used for juicing and determination of the content of TSS (*w*/*w*, %), TA (*w*/*v*, %), and VC.

### 2.6. Determination of Mass Loss, Pericarp Relative Conductivity, Pericarp Browning Index, and Aril Breakdown Index

Inner pericarp browning index and aril breakdown index were estimated according to a previously reported method [3,7]. Before sampling, the inner pericarp browning index was assessed by measuring the extent of the total browned area (TBNA) on pericarp of fruits from each tray: score = 0, no browning; score = 0.01 to 1.00, TBNA was 0–20%; score = 1.01 to 2.00, TBNA was 20.1–40%; score = 2.01 to 3.00, TBNA was 40.1–60%; score = 3.01 to 4.00, TBNA was 60.1–80%; score = 4.01 to 5.00, TBNA was 80.1–100%. The pericarp browning index of each tray was calculated as follows:

Pericarp browning index = ∑ (TBNA score of each fruit)/(total evaluated fruits)(1)

The aril breakdown index was assessed by measuring the extent of the total breakdown area (TBDA) on each longan aril using the following scales: score = 0, no breakdown; score = 0.01 to 1.00, TBDA was 0–25%; score = 1.01 to 2.00, TBDA was 25.1–50%; score = 2.01 to 3.00, TBDA was 50.1–75%; score = 3.01 to 4.00, TBDA was 75.1–99.9%; score = 5, TBDA was 100%. The aril breakdown index of each bag was calculated using Formula (2):

Aril breakdown index = ∑ (TBDA score of each fruit)/(total evaluated fruits) (2)

### 2.7. Determination of TSS, TA, Vitamin C, and Reduced Glutathione (GSH) in Postharvest Longan Samples

The TSS content (*w/w*, %) and TA content (*w*/*v*, %) were measured by a Brix refractometer (PAL-BX/ACID F5, ATAGO Co., Ltd., Tokyo, Japan). The VC content (in juice, g L^−1^; in pericarp, mg g^−1^) of fruits was determined by an ultraviolet–visible spectrophotometry method. A volume of 10 μL longan juice was diluted with 9900 μL oxalic acid solution (2%, *w/v*, pH 6.0 adjusted with NaOH) and then the absorbance at 267 nm was measured. The VC content was calculated using a standard curve. The extract used for determination of GSH content was prepared by homogenizing 0.1 g of pericarp powder with 900 µL PBS buffer (50 mM, pH 7.0) or 0.2 g of pulp powder with 800 µL PBS buffer. After being fully vortexed, the sample was subjected to centrifugation at 2500× *g* and 4 °C for 10 min [30]. The contents of GSH of longan pericarp and pulp were measured based on reaction with DTNB by using a commercialized GSH assay kit (A006-2-1) from Nanjing Jiancheng Bioengineering Institute (www.njjcbio.com/products.asp?id=1532 (accessed on 5 October 2020)). The absorbance of each sample was measured at 405 nm and the GSH content was calculated according to a standard curve.

### 2.8. Statistical Analysis

The results obtained were expressed as the mean ± SE of four randomized replicates. The data were subjected to analysis of variance (ANOVA). The statistical significance between the control and the treated sample was analyzed by paired-samples t-tests, while the multiple comparison was performed by one-way analysis of variance based on Duncan’s multiple range tests, using SPSS version 17 (IBM Corp., Armonk, NY, USA).

## 3. Results and Discussion

### 3.1. GA_3_ Treatment Affects TSS, Antioxidants, and Related Enzyme Activities in Longan Fruit during Ripening and On-Tree Preservation

**Figure 1:**
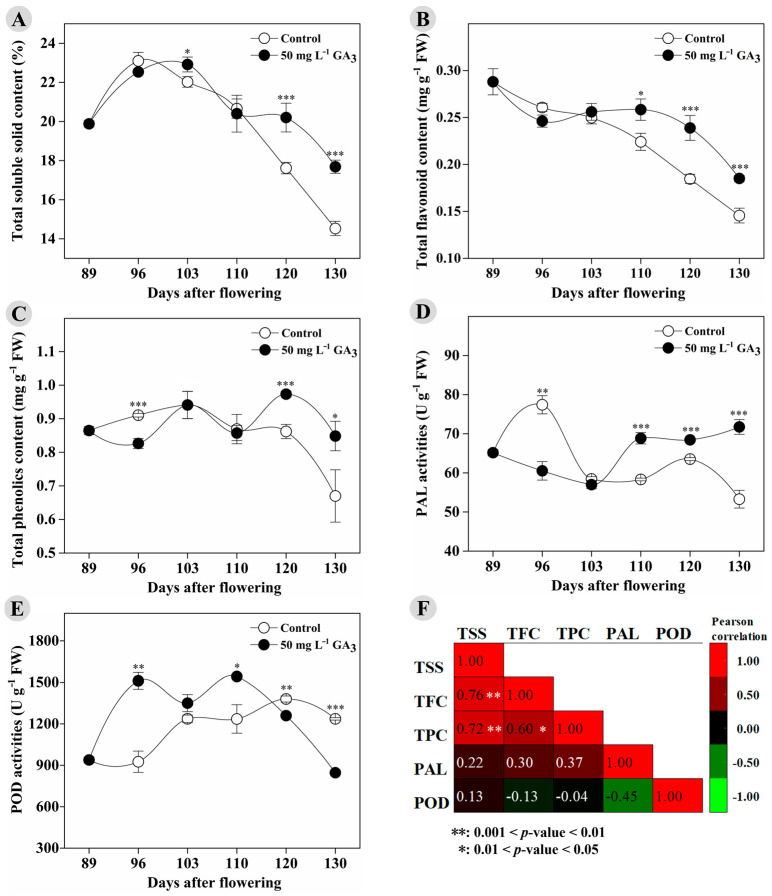
The changes in total soluble solids content, antioxidant total content, and related enzyme activities in the control and GA_3_-treated longan fruit during ripening and on-tree preservation stages: (**A**) total soluble solid content; (**B**) total flavonoid content; (**C**) total phenolics content; (**D**) phenylalanine ammonia-lyase activities; (**E**) peroxidase activities; (**F**) correlation between the indicators. Samples used in this analysis were harvested in year 2020. * 0.01 ≤ *p*-value < 0.05; ** 0.001 ≤ *p*-value < 0.01; *** *p*-value < 0.001.

Through a set of GA_3_ treatments with different concentrations for 2 years, the results suggested that 50 mg L^−1^ GA_3_ had a significant effect on delaying decline of TSS (sugar receding) (Figure 1A and Figure S1). In the experiment of year 2020, 10 mg L^−1^, 20 mg L^−1^, and 50 mg L^−1^ GA_3_ had no significant effect on the TSS content at ripening and harvest stages (96–110 DAF), but 50 mg L^−1^ GA_3_ significantly reduced the degree of TSS decline at the later on-tree preservation stages (120 DAF, 11.96% higher; 130 DAF, 22.00% higher than the control) (Figure S1A). Further, the results from year 2021 also showed that 50 mg L^−1^ GA_3_ had a better effect than 10 and 150 mg L^−1^ GA_3_ on delaying the decline of TSS at 105 to 130 DAF (Figure S1B).

The 50 mg L^−1^ GA_3_ treatment also showed an effect to maintain the content of antioxidants (mainly phenolics and flavonoids) and the activity of PAL, a key rate-limited enzyme for synthesis of phenolic acids and flavonoids during the later on-tree stages (Figure 1B–D). The above-mentioned results of TFC and TPC might be explained by the pattern of changes in PAL activity, which was lower at 96 DAF but higher at 110, 120, and 130 DAF in the GA_3_-treated pulp compared to the control pulp (Figure 1D). It was worth noting that the POD activity in the GA_3_-treated pulp was significantly higher at 96 and 110 DAF, but lower at 120 and 130 DAF (Figure 1E). The Pearson correlation analysis indicated that TSS content showed a significant positive correlation with TFC (r^2^ = 0.76, *p* = 0.0044) and TPC (r^2^ = 0.7, *p* = 0.0077). A significant positive correlation was also found between TFC and TPC (r^2^ = 0.60, *p* = 0.0395; Figure 1F). These results indicated that the GA_3_ treatment significantly affected total soluble solids, antioxidants, and related enzyme activities in longan fruit during ripening and on-tree preservation. In recent years, preharvest or postharvest GAs application has attracted much attention in postharvest technology of fruits, due to their positive regulation of fruit size [13,16,17,18,19,20], firmness [16,20], anthocyanin content [11], and PAL activity [11], as well as inhibition effect on respiration rate [15,20], postharvest ripening [15], and deterioration [9,15]. In this study, we found that preharvest GA_3_ treatment with an appropriate concentration (50 mg L^−1^) led to decreased TPC, TFC, and PAL activity (similar to the results reported in the GA_3_-treated grape berry skin and flesh [13] and cherry [17]) in longan pulp at the mature stage. However, the treatment showed a significant effect to delay the sugar receding and maintain a higher sweetness (the most important commodity-related property of longan [3]). Moreover, the treatment enhanced the accumulation of antioxidants and PAL activity in pulp during the on-tree preservation.

### 3.2. Identification, Quantification, and Classification of Metabolites Detected in the GA_3_-Treated and Control Longan Pulp

**Figure 2:**
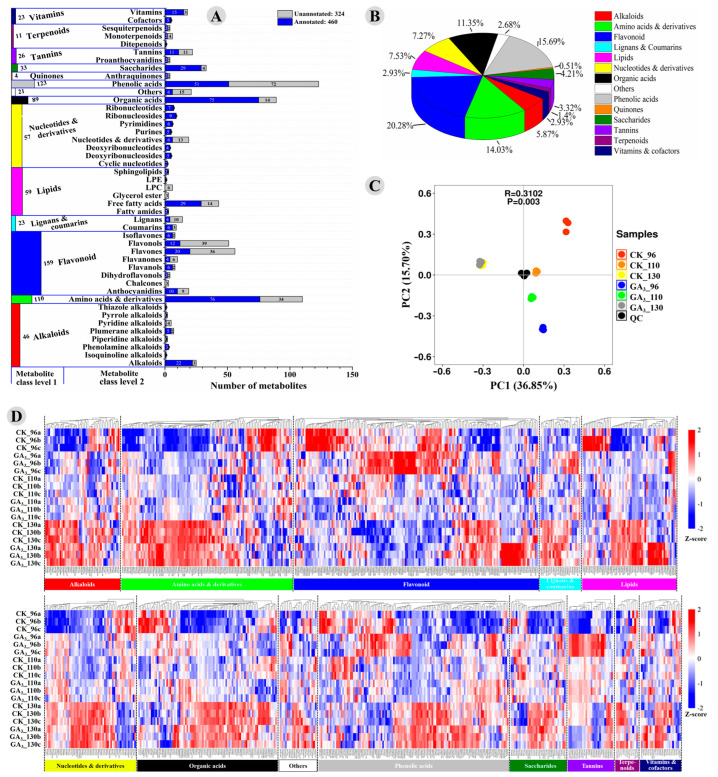
Statistics of the metabolites detected in the control and the GA_3_-treated longan pulp: (**A**) statistics of the detected and annotated metabolites in each class; (**B**) percentage of the metabolites in each class; (**C**) PCA analysis of the control and GA_3_-treated longan pulp samples at 96, 110, and 130 DAF; (**D**) cluster analysis of metabolites in each class. CK: Control; GA_3_: 50 mg L^−1^ GA_3_.

In order to systematically reveal the effect of GA_3_ treatment on the metabolic profile of ‘Shixia’ longan pulp, a widely targeted metabolome by HPLC-ESI-triple quadrupole-linear ion trap (QTRAP)–MS was used to identify and quantify the metabolites. In total, 784 metabolites categorized into 14 classes (level 1) were detected in the ‘Shixia’ longan pulp (Figure 2A). The metabolome consisted of 46 alkaloids (5.87%), 110 amino acids and derivatives (14.03%), 159 flavonoid (20.28%), 23 lignans and coumarins (2.93%), 59 lipids (7.53%), 57 nucleotides and derivatives (7.27%), 89 organic acids (11.35%), 21 others (belong to no clear class) (2.68%), 123 phenolic acids (15.69%), 4 quinones (anthraquinones) (0.51%), 33 saccharides (4.21%), 26 tannins (3.32%), 11 terpenoids (1.4%), and 23 vitamins (2.93%) (Figure 2A,B). Among the 784 detected metabolites, 460 metabolites were annotated with compound ID and 324 metabolites were not annotated in the KEGG compound database (Figure 2A). It was worth noting that more metabolites (784) were identified in this work compared to those in the previously reported analyses of ‘Shixia’ longan pulp (514 [2]; 706 [31]).

The principal component analysis (PCA) results indicated that the GA_3_-treated samples at 96 DAF (CK_96) and 110 DAF (CK_110) were significantly different from the control samples at 96 (GA_3__96) and 110 DAF (GA_3__110). However, the GA_3_-treated samples at 130 DAF (CK_130) were almost overlapped with the control samples at 130 DAF (GA_3__130) (Figure 2C). These results indicated the significant difference between the control and GA_3_-treated samples, especially the samples from 96 DAF and 110 DAF. A cluster heatmap gave a global view of each class of metabolites. Significant difference was found within each class of metabolites between the control and the GA_3_-treated samples, especially at 96 DAF (Figure 2D). At 96 DAF, more than 60% of the metabolites from the following 9 classes were up-regulated in the GA_3_-treated pulp: amino acids and derivatives (67/110, 60.91%), lignans and coumarins (18/23, 78.26%), nucleotides and derivatives (39/57, 68.42%), Others (13/21, 61.90%), phenolic acids (76/123, 61.79%), saccharides (29/33, 87.88%), tannins (26/26, 100%), and vitamins and cofactors (16/23, 69.57%) (Figure S2A). At 110 DAF, 73.08% of tannins were up-regulated in the GA_3_-treated pulp (Figure S2B). However, 65.38% of the tannins were down-regulated in the GA_3_-treated pulp at 130 DAF (Figure S2C). The above results indicated that the secondary metabolic profile, especially the composition and content of antioxidants, in the on-tree preserved longan pulp was profoundly changed by the 50 mg L^−1^ GA_3_ treatment. Although higher TFC and TPC were found in the 50 mg L^−1^ GA_3_-treated pulp at 110 DAF and 130 DAF, the effect of the treatment on the metabolic profile of longan pulp became weaker with the extension of on-tree preservation (Figure 2C,D and Figure S2B,C).

### 3.3. Significantly Differently Accumulated Metabolites between the GA_3_-Treated and Control Longan Pulp

**Figure 3:**
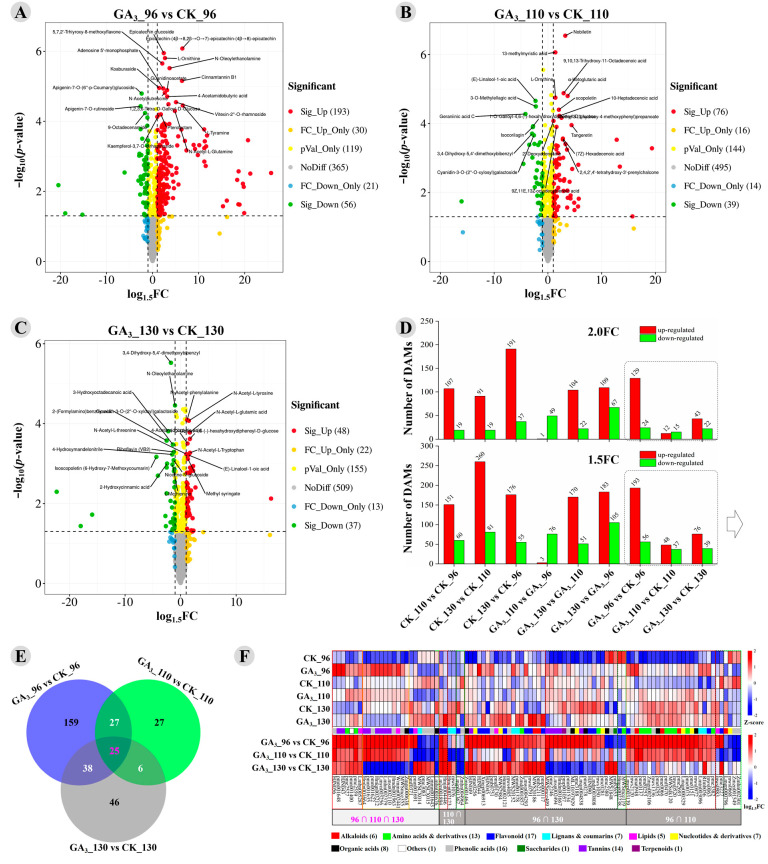
The statistics of the DAMs in the GA_3_-treated longan pulp compared to the control at 96, 110, and 130 DAF. Volcano plot maps of DAMs from (**A**) the GA_3__96 vs. CK_96, (**B**) GA_3__110 vs. CK_110, and (**C**) GA_3__130 vs. CK_130. (**D**) Number of DAMs at 1.5-fold or 2-fold change; (**E**) Venn diagram of DAMs (1.5-FC) of the three stages; (**F**) heatmap of the common DAMs from at least two stages. CK: Control; GA_3_: 50 mg L^−1^ GA_3_.

Based on the OPLS-DA analysis and significance test, 249 DAMs with 1.5-fold change (FC), containing 193 up-regulated and 56 down-regulated metabolites, were found in GA_3__96 compared to CK_96 (Figure 3A,D); moreover, 115 DAMs with 1.5-FC containing 76 up-regulated and 56 down-regulated metabolites were observed in GA_3__110 compared to CK_110 (Figure 3B,D), but only 85 DAMs with 1.5-FC containing 48 up-regulated and 37 down-regulated metabolites were observed in GA_3__130 compared to CK_130 (Figure 3C,D). Many more DAMs with 2.0-FC (126 DAMs) or 1.5-FC (211 DAMs) were found when comparing the CK_110 vs. CK_96 than were found for the DAMs from the GA_3__110 vs. GA_3__96 (50 DAMs with 2.0-FC; 79 DAMs with 1.5-FC); similar results were observed in the DAMs from the CK_130 vs. CK_96 compared to those from the GA_3__130 vs. GA_3__96 (Figure 3D).

A Venn analysis of DAMs with 1.5-FC from the GA_3__96 vs. CK_96, GA_3__110 vs. CK_110, and GA_3__130 vs. CK_130 indicated that 25 metabolites—including 2 amino acids, 1 flavonoid, 1 coumarin, 3 lipids, 2 organic acids, 7 phenolic acids, and 8 tannins—were all significantly differently accumulated in the GA_3_-treated longan pulp at 96 DAF, 110 DAF, and 130 DAF compared to the control longan pulp (Figure 3E,F). Moreover, 27 metabolites were found to be significantly differently accumulated in the GA_3_-treated longan pulp at both 96 DAF and 110 DAF compared to the control longan pulp; 6 metabolites were found to be significantly differently accumulated in the GA_3_-treated longan pulp at both 110 DAF and 130 DAF compared to the control longan pulp; and 38 metabolites were significantly differently accumulated in the GA_3_-treated longan pulp at both 96 DAF and 130 DAF compared to the control longan pulp (Figure 3E,F). Therefore, 96 metabolites were found to be significantly differently accumulated in the GA_3_-treated longan pulp at 2 or 3 stages compared to the control longan pulp (Figure 3E). A heatmap of these 96 common DAMs showed their expression pattern. The 96 common DAMs were composed of 6 alkaloids, 13 amino acids and derivatives, 17 flavonoid, 7 lignans and coumarins, 5 lipids, 7 nucleotides and derivatives, 8 organic acids, 1 other, 16 phenolic acids, 1 saccharide, 14 tannins. and 1 terpenoid (Figure 3F). The above results indicated that the GA_3_ treatment had a significant influence on the flavonoids (17/159), lignans and coumarins (7/23), phenolic acids (16/123), and tannins (14/26) which were related to antioxidant ability in the longan pulp during the on-tree preservation especially at 96 DAF and 110 DAF (Figure S3). The above result was consistent with the reported positive effect of GA_3_ treatment on the content of phenolics in jujube fruit [20], but contrary to the results of TFC and TPC reported in the grape berry flesh [13] and TPC reported in the cherry fruit [17]. Therefore, the influence of exogenous GA_3_ treatment on the antioxidants depends on the fruit species, edible organ, treatment dose, time, and other factors.

### 3.4. KEGG Enrichment of DAMs and Display of Important Metabolic Pathways

**Figure 4:**
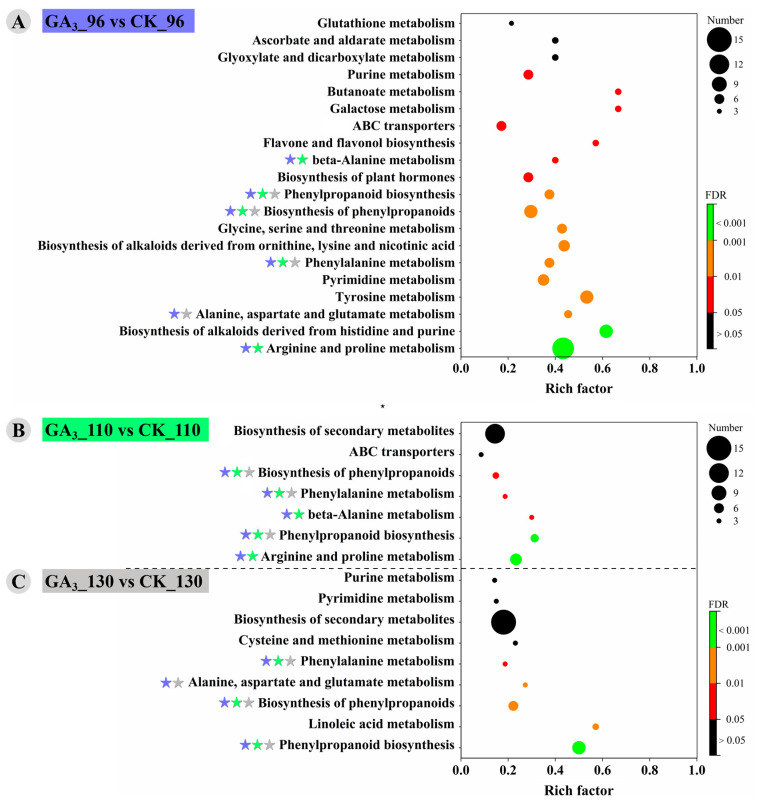
KEGG enrichment analysis of the DAMs from (**A**) the GA_3__96 vs. CK_96, (**B**) GA_3__110 vs. CK_110, and (**C**) GA_3__130 vs. CK_130. The star marks represent common enriched pathways—blue star: from the GA_3__96 vs. CK_96; green star: from the GA_3__110 vs. CK_110; gray star: from the GA_3__130 vs. CK_130. CK: Control; GA_3_: 50 mg L^−1^ GA_3_.

KEGG enrichment analysis showed that the DAMs from the GA_3__96 vs. CK_96 were significantly enriched (FDR < 0.05) in 17 pathways, including arginine and proline metabolism; biosynthesis of alkaloids derived from histidine and purine; alanine, aspartate and glutamate metabolism; tyrosine metabolism; pyrimidine metabolism; phenylalanine metabolism; biosynthesis of alkaloids derived from ornithine, lysine and nicotinic acid; glycine, serine and threonine metabolism; biosynthesis of phenylpropanoids; phenylpropanoid biosynthesis; biosynthesis of plant hormones; beta-alanine metabolism; flavone and flavonol biosynthesis; ABC transporters; galactose metabolism; butanoate metabolism; and purine metabolism (Figure 4A and Figure S3). The DAMs from the GA_3__110 vs. CK_110 were significantly enriched in five pathways, including arginine and proline metabolism, phenylpropanoid biosynthesis, β-alanine metabolism, and phenylalanine metabolism (Figure 4B). Moreover, phenylpropanoid biosynthesis, linoleic acid metabolism, alanine, aspartate and glutamate metabolism, and phenylalanine metabolism were significantly enriched for the GA_3__130 vs. CK _130 (Figure 4C). It was interesting to note that the DAMs from the GA_3__96 vs. CK_96, GA_3__110 vs. CK_110, and GA_3__130 vs. CK_130 were commonly enriched in phenylalanine metabolism and biosynthesis of phenylpropanoids.

### 3.5. The Effect of Preharvest 50 mg L^−1^ GA_3_ Treatment on Storability and Postharvest Fruits’ Antioxidants during Room-Temperature Storage

**Figure 5:**
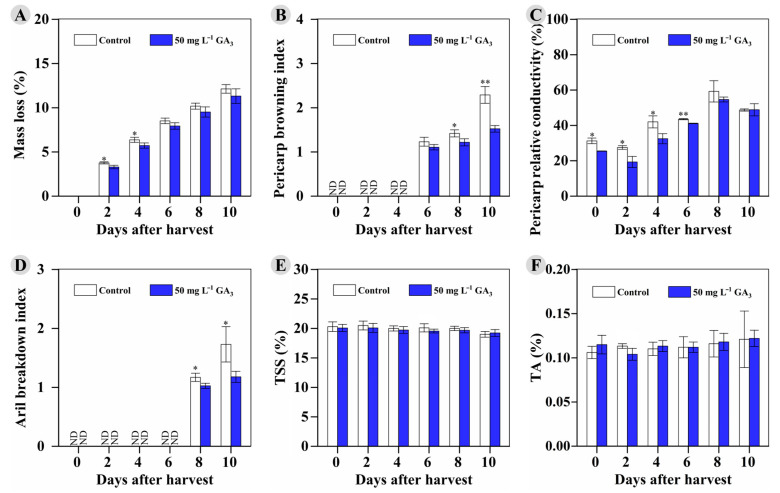
The changes in indexes related to storability and quality of the control and 50 mg L^−1^ GA_3_-treated fruits during RT storage: (**A**) mass loss; (**B**) pericarp browning index; (**C**) pericarp relative conductivity; (**D**) aril breakdown index; (**E**) TSS content (%); (**F**) TA content (%). * 0.01 ≤ *p*-value < 0.05; ** 0.001 ≤ *p*-value < 0.01.

In addition to the reported effect of GA_3_ on fruit quality (strawberry [11], ‘French’ prune [12], grape berry [13], pear fruit [14], cherry [17], sour cherry [18], “Washington Navel” orange [19], jujube fruit [20]) at harvest, as well as the maintenance of VC and phenolic substances after harvest, preharvest treatments with oxalic acid (on sweet cherry [32] and lemon fruit [33]), melatonin (on pomegranate [34]), and methyl jasmonate (on plum [35]) were proven to improve fruit quality and storability, stimulate the antioxidant system, and enhance accumulation of bioactive compounds. In this study, the longan fruits for the storage experiment were sprayed with 50 mg L^−1^ GA_3_ or the control solution 2 times (at 85 DAF and 95 DAF) and harvested at 105 DAF. Our results showed that 50 mg L^−1^ GA_3_ significantly increased the storability of ‘Shixia’ longan fruit by delaying mass loss, pericarp browning, increase in relative conductivity, and aril breakdown during the RT storage (Figure 5A–D). As shown in Figure 5A, lower total mass loss (%) was found in the treated longan fruit throughout the storage (significant at 2 DAH and 4 DAH). More importantly, significantly delayed development of pericarp browning and lower relative conductivity (significant at 0 DAH to 6 DAH) were observed in the treated longan fruit (Figure 5B,C). In addition, the aril breakdown index of the treated longan was significantly lower than that of the control, which developed quickly at the two later stages (8 DAH and 10 DAH) (Figure 5D). However, the 50 mg L^−1^ GA_3_ treatment did not significantly influence the TSS and TA content of ‘Shixia’ longan at harvest. No significant difference in TSS and TA content was observed between the treated longan and the control throughout storage (Figure 5E,F). The above results indicated that the 50 mg L^−1^ GA_3_ treatment significantly improved the storability of ‘Shixia’ longan fruit.

**Figure 6:**
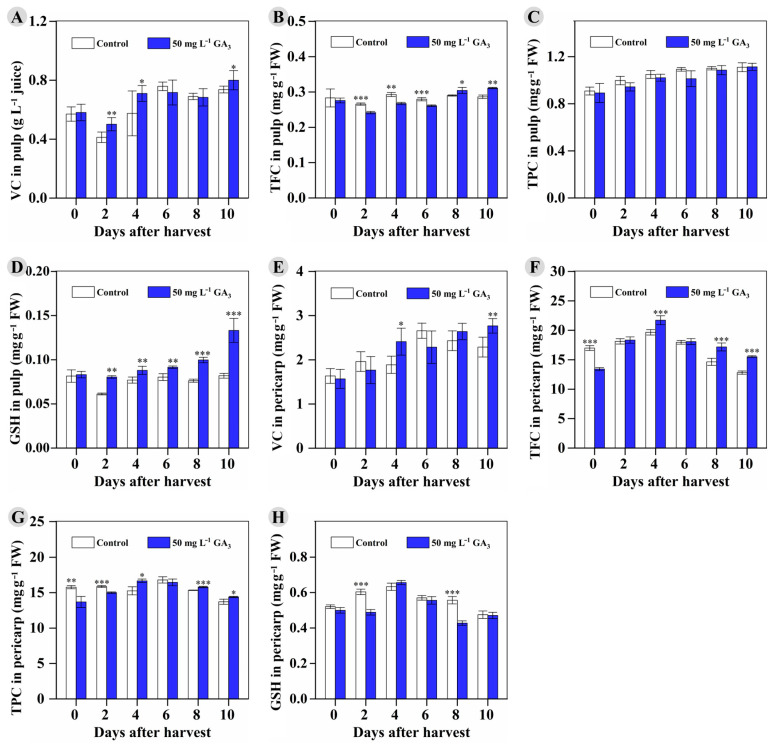
The vitamin C, total flavonoid content, total phenolic content, and GSH content in the control and 50 mg L^−1^ GA_3_-treated fruits during RT storage. (**A**–**D**): pulp; (**E**–**H**): pericarp. * 0.01 ≤ *p*-value < 0.05; ** 0.001 ≤ *p*-value < 0.01; *** *p*-value < 0.001.

Similar effects as reported in previous works [32,33,34,35] were also observed in the GA_3_ treatment on longan samples: significantly higher VC content was detected in the treated longan pulp at 2, 4, and 10 DAH (Figure 6A). Lower TFC at the earlier stages of storage (2 DAH, 4 DAH, and 6 DAH) but significantly higher TFC at 8 DAH and 10 DAH were observed in the treated longan pulp compared to that in the control (Figure 6B). The TPC slowly increased along with the RT storage time and showed no difference between the treated longan pulp and the control pulp (Figure 6C). Interestingly, the GSH content in the treated longan pulp increased and was significantly higher than that in control pulp during the entirety of RT storage (2 DAH to 10 DAH) (Figure 6D).

The VC content in the control longan pericarp increased from 0 DAH to 6 DAH, and then slowly decreased. Significantly higher VC was observed at 4 DAH and 10 DAH in the GA_3_-treated pericarp (Figure 6E). The TFC in the control longan pericarp was significantly higher than that in the treated pericarp at harvest (0 DAH), but it decreased rapidly from 4 DAH to 10 DAH. However, the TFC in the treated longan pericarp was significantly higher than that in the control at the later storage stages (4, 8, and 10 DAH) (Figure 6F). Similarly, the TPC in the treated pericarp was significantly lower than that in the control pericarp at the earlier stages of storage (0 DAH and 2 DAH), but it was significantly higher than that in the control at 4, 8, and 10 DAH (Figure 6G). Moreover, the GSH content in the control longan pericarp increased from 0 to 4 DAH, but then decreased. Lower GSH content was observed in the treated longan pericarp at only 2 and 8 DAH (Figure 6H). In total, the 50 mg L^−1^ GA_3_ treatment significantly enhanced the accumulation of VC in longan pericarp and pulp and GSH in pulp at the later storage stages, or helped maintain the TFC in pulp and both the TPC and TFC in pericarp. A similar effect was reported in the literature, that the combined treatments of CPPU and GA_3_ on green mature banana significantly delayed the loss of ascorbic acid and total phenols, retarding chlorophyll degradation during storage [15].

Many previous works indicated that longan pericarp browning was related to phenolic metabolism [36], and the maintenance of flavonoids, phenolics, and other antioxidants (i.e., VC and GSH) helped balance the scavenging ability and production of oxygen free radicals [26,30,36], which protected cytomembrane integrity [7] and, thus, inhibited pericarp browning. Flavonoids have been proven as essential barriers against pathogen attack [37]. In addition, higher levels of VC, flavonoids, GSH, and total phenolics, as well as higher ability of scavenging free radicals, have been proven to help maintain the structural integrity of longan pulp cell membrane and suppress the pulp breakdown occurrence in postharvest longan fruit [38]. Our results indicated that the 50 mg L^−1^ GA_3_ treatment significantly improved the storability of ‘Shixia’ longan fruit, possibly through maintaining or increasing the antioxidant content at the later storage stages but not at harvest.

## 4. Conclusions

Preharvest GA_3_ treatment with an appropriate concentration (50 mg L^−1^) showed a significant effect in delaying the sugar receding of ‘Shixia’ longan, a very important main variety in China. Moreover, higher content of antioxidant bioactive compounds (mainly phenolic acids, flavonoids, tannins, lignans, and coumarins) and higher PAL activity were found in the GA_3_-treated pulp during the on-tree preservation stages. More importantly, 50 mg L^−1^ GA_3_ did not influence the TSS and TA content, but significantly delayed pericarp browning and aril breakdown, which resulted in lower pericarp relative conductivity and mass loss but higher vitamin C and TFC, along with reduced glutathione in pulp and higher vitamin C, TFC, and TPC in pericarp at the later RT storage stages. Overall, these results confirmed that preharvest 50 mg L^−1^ GA_3_ spraying could be a practical tool to delay the sugar receding of longan fruit, and thus prolong its harvest time and supply time as fresh fruit, as well as enhance antioxidant compounds, improve functional properties, and reduce postharvest loss during RT storage.

## Data Availability

The data presented in this review are available in the article.

## Supplementary Materials

The following supporting information can be downloaded at: https://www.mdpi.com/article/10.3390/foods12102032/s1, Figure S1. The TSS content of the GA_3_-treated and control longan pulp in the year 2020 and 2021; Figure S2. Statistics of metabolites in each class according to the fold change by the GA_3_ vs. control longan pulp; Figure S3. The fold changes of the metabolites from the important pathways. CK: Control; GA_3_: 50 mg L^-1^ GA_3_.
