# Supplementary material for: GA3 Treatment Delays the Deterioration of ‘Shixia’ Longan during the On-Tree Preservation and Room-Temperature Storage and Up-Regulates Antioxidants
Source: Foods. 2023 May 17;12(10):2032. doi: 10.3390/foods12102032 (PMC10217209; doi:10.3390/foods12102032)
Supplement: Supplementary file 1 [file foods-12-02032-s001.zip › foods-2366860-supplementary.pdf]

foods
MDPI

Article

GA_3 Treatment Delays the Deterioration of 'Shixia' Longan during the On-Tree Preservation and Room-Temperature Storage and Up-Regulates Antioxidants

Tao Luo [1,2,3,†], Xiaolan Lin [1,2,†], Tingting Lai [1,2], Libing Long [1,2], Ziying Lai [1,2], Xinxin Du [1,2], Xiaomeng Guo [1,2], Liang Shuai [4], Dongmei Han [5] and Zhenxian Wu [1,2,3,*]

1 College of Horticulture, South China Agricultural University, Guangzhou 510642, China; luotao0502@scau.edu.cn (T.L.); linxl4595@stu.scau.edu.cn (X.L.); ltt7059@stu.scau.edu.cn (T.L.); longlibing@stu.scau.edu.cn (L.L.); laiziying@stu.scau.edu.cn (Z.L.); xinxindu@stu.scau.edu.cn (X.D.); 31000462@scau.edu.cn (X.G.)
2 Guangdong Provincial Key Laboratory of Postharvest Science of Fruits and Vegetables, Engineering Research Center of Southern Horticultural Products Preservation, Ministry of Education, Guangzhou 510642, China
3 Key Laboratory of Biology and Genetic Improvement of Horticultural Crops (South China), Ministry of Agriculture and Rural Affairs, College of Horticulture, South China Agricultural University, Guangzhou 510642, China
4 College of Chemistry and Food Science, Nanchang Normal University, Nanchang 330032, China; shuailiang1212@hzxy.edu.cn
5 Institute of Fruit Tree Research, Guangdong Academy of Agricultural Sciences, Key Laboratory of South Subtropical Fruit Biology and Genetic Resource Utilization, Ministry of Agriculture, Guangzhou 510640, China; handongmei@gdaas.cn
* Correspondence: zhenxwu@scau.edu.cn.
† These authors contributed equally to this work.

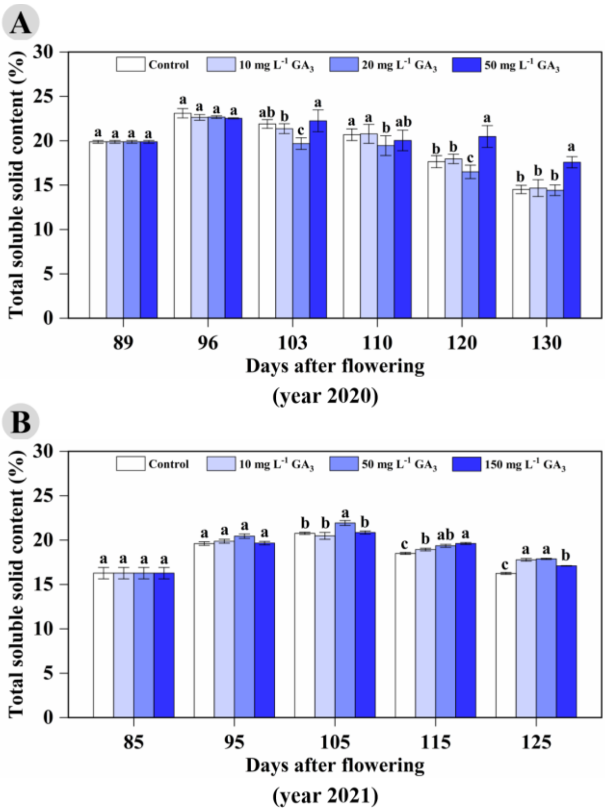

Figure S1. The TSS content of the GA_3 treated and control longan pulp in the year 2020 and 2021.

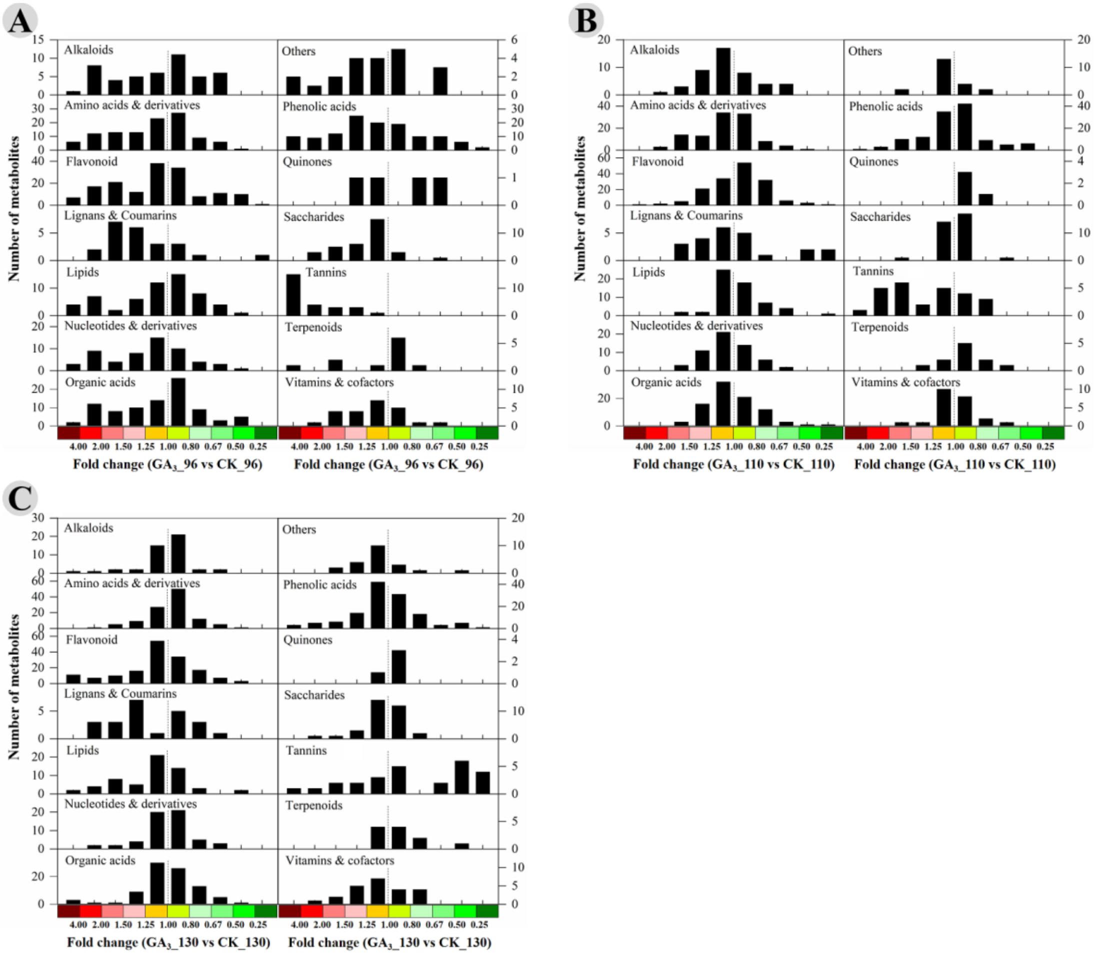

Figure S2. Statistics of metabolites in each class according to the fold change by the GA_3 vs. control longan pulp.

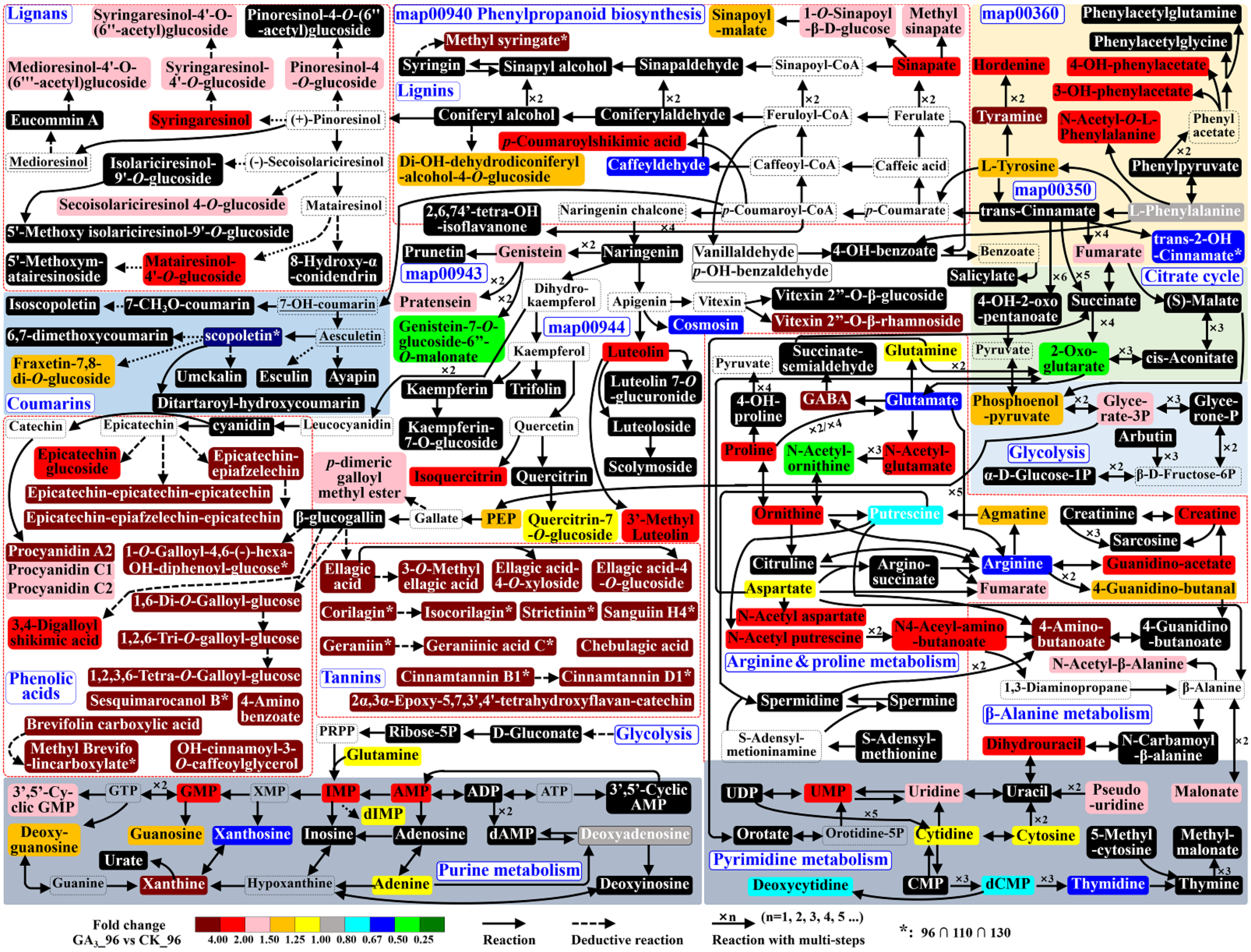

Figure S3. The fold changes of the metabolites from the important pathways. CK: Control; GA_3: 50 mg L^{-1} GA_3.
